# Endothelial Progenitor Cells in Moyamoya Disease: Current Situation and Controversial Issues

**DOI:** 10.1177/0963689720913259

**Published:** 2020-03-20

**Authors:** Jin Yu, Qian Du, Miao Hu, Jianjian Zhang, Jincao Chen

**Affiliations:** 1 Department of Neurosurgery, Zhongnan Hospital of Wuhan University, Wuhan, China; 2 Department of Rheumatology, Xiangya Hospital, Central South University, Changsha, Hunan, China

**Keywords:** endothelial progenitor cells, moyamoya disease, neovascularization, pathogenesis

## Abstract

Due to the lack of animal models and difficulty in obtaining specimens, the study of pathogenesis of moyamoya disease (MMD) almost stagnated. In recent years, endothelial progenitor cells (EPCs) have attracted more and more attention in vascular diseases due to their important role in neovascularization. With the aid of paradigms and methods in cardiovascular diseases research, people began to explore the role of EPCs in the processing of MMD. In the past decade, studies have shown that abnormalities in cell amounts and functions of EPCs were closely related to the vascular pathological changes in MMD. However, the lack of consistent criteria, such as isolation, cultivation, and identification standards, is also blocking the way forward. The goal of this review is to provide an overview of the current situation and controversial issues relevant to studies about EPCs in the pathogenesis and etiology of MMD.

## Introduction

Moyamoya disease (MMD) is an idiopathic cerebrovascular disease which was first described by Suzuki and Takaku in 1969^1^. MMD is characterized by progressive stenosis or occlusion at the end of bilateral internal carotid artery (ICA) and/or the beginning of anterior and middle cerebral artery, accompanied by compensatory dilation of the perforating artery, and formation of dense vascular networks (“moyamoya vessels”)^2^. MMD has been found all over the world, especially in Japan, Korea, and China^3^. At present, the treatment of MMD is mainly based on revascularization surgery^4^ which is just a late-stage intervention considering the long development process of this disease. Due to the lack of clear understanding of its etiology and pathogenesis, there is almost no way to carry out any early prevention and intervention for MMD.

Recently, increasing attention has been paid to the important role of endothelium in cerebrovascular biology^5–7^. In fact, several vascular pathological changes, such as intima hyperplasia, tortuous layering of internal elastic lamina, and abnormal angiogenesis, have already been observed in MMD^8^. Therefore, the potential role of endothelial progenitor cells (EPCs) in the pathogenesis of MMD has aroused the interest of researchers, especially in maintaining endothelial integrity, function, and postnatal neovascularization. With the help of paradigms provided by studies on EPCs in other vascular diseases (e.g., cardiovascular disease^9^, cerebral ischemic stroke^10^), especially the process involving neovascularization, the exploration of EPCs in the mechanism of MMD has made progress in the past decade.

The goal of this review is to provide an overview of the current situation and controversial issues relevant to studies about EPCs in the pathogenesis and etiology of MMD.

## What Is Neovascularization?

Neovascularization, the process of new blood vessel growth and development, is an important process under various physiological and pathological conditions such as embryonic development^11^, wound healing^12^, ischemia, inflammation, infections^13^, as well as tumorigenesis^14^. The molecular, genetic, and cellular mechanisms of vessel growth and their implications are not always the same under different circumstances. Over the years, many related studies have developed the concept of the genesis of new vessels into some similar terms with different connotations.

The term “angiogenesis” describes the initiation of new capillaries from preexisting vessels^13^, which are stimulated by hypoxia through the activation of numerous growth factors and cytokines^15^. *Arteriogenesis* refers to the maturation or regrowth of collateral vessels^16^, which are usually large enough to be shown on angiography^17^. Arteriogenesis usually occurs outside the ischemic area in response to the aggregation of blood-derived monocytes in localized arterial stenosis site caused by local shear stress. One of the most important arguments related to arteriogenesis is whether collateral development occurs similar to angiogenesis or it represents the remodeling and dilation of preexisting vascular channels^14^. *Vasculogenesis* is an important paradigm for the establishment of embryonic primitive vascular network^18^, a process of vascular formation in situ by circulating EPCs and vascular progenitor cells^19,20^. In contrary, “postnatal vasculogenesis” refers to new blood vessel formation in adults^21^. And the last, *neovascularization* is the result of several processes, including angiogenesis, arteriogenesis, and vasculogenesis^14^.

It should be noted that these processes are not completely independent; for example, in the case of a common femoral artery ligation, arteriogenesis will predominate at the site of ligation, whereas angiogenesis will predominate in the ischemic distal bed^14^. Therefore, it is necessary to study a particular neovascularization event according to the specific pathological conditions in specific diseases.

## EPCs in Postnatal Neovascularization

EPCs were first discovered in isolated mononuclear cells (MNCs) from human peripheral blood (PB) by Asahara et al. in 1997^22^. These bone marrow (BM)–derived progenitor cells with high proliferative ability were defined as EPCs, which have the potential to differentiate into endothelial cells (ECs) lines^23^. EPCs were found to be involved in the physiological process of neovascularization like wound healing and ovarian cycle and subsequent pathological events, such as hypertension^24^, myocardial infarction^25^, stroke^26^, atherosclerosis^27^, and cancer^28^.

There have been controversies about the origin of EPCs. At present, it is generally accepted that EPCs originated from mesoderm cells, the same origination as hematopoietic stem cells (HSCs), during embryonic development^29^. Normally, EPCs retain in a homeostatic BM microenvironment with low oxygen tension and high stromal cell–derived factor-1 (SDF-1) content, which is necessary for maintaining them^30^. Stimulated by factors such as inflammatory, traumatic, or ischemia-induced hypoxia, EPCs leave the BM and enter the circulation driven by chemokines, matrix metalloproteinase (MMP) 9, vascular endothelial growth factor (VEGF), nitric oxide, and so on, which is called “mobilization”^31,32^. Regulated by tissue-specific chemokine signaling, EPCs become activated and home to the target tissue. On reaching the site of injury, EPCs begin to adhere to ECs and migrate into vascular and tissue repair sites under the mediation of integrins^33^. Once EPCs pass through the endodermis, they perform their function by differentiating into ECs and remodeling the vascular extracellular matrix (ECM) components^34,35^. Although the functional activity of EPCs is mostly under investigation, it is considered that their differentiation involves adhesion to the ECM components controlled by integrins, proliferation and survival induced by growth factors, and maturation and acquisition of the endothelial phenotype^36^.

EPCs also contribute to the maintenance of the vascular system by producing proangiogenic factors able to enhance the proliferation, survival, and function of mature ECs and other surrounding progenitor cells^23^. For example, smooth muscle progenitor cells (SMPCs) and smooth muscle cells (SMCs) are key factors in proliferative vascular diseases such as atherosclerosis, intimal hyperplasia, and hypertension^37^. Studies have found that EPCs were closely related to the source and function of SMPC and SMC^38–41^. Besides, the abnormalities in the amount and functions of EPCs were also found in chronic ischemic cardiomyopathy^42^, myocardial infarction^25^, ischemic stroke^10^ and infarct models^43,44^, which prompted the participation of EPCs in vascular occlusive/stenosis process.

As mentioned earlier, EPCs share common precursors (mesoderm cells) with other cell lineages. Therefore, it is feasible to separate EPCs from various sources, such as hematopoietic EPCs (hemogenic endothelium, myeloid cells, mesenchymal stem cells), nonhematopoietic EPCs (umbilical cord blood, PB), and tissue-resident EPCs^45^. EPCs derived from PB, also known as circulation EPCs (cEPCs), have been studied most because the method to obtain specimens is more convenient and less invasive. A set of methods created by Asahara et al.^22^ and developed by later researchers were used to isolate and cultivate the cEPCs^46^. Accordingly, two different types of circulating EPCs (early EPCs and late EPCs) have been identified according to their morphology, appearance time, and cell surface markers^47–49^. The specific differences between these two kinds of EPCs will be elaborated in the later review.

## Abnormal Neovascularization in MMD

According to the definition of MMD, there are at least two impaired vessel growth processes in the course of this disease. The first is the proliferative lesions that cause stenosis/occlusion in major cerebral arteries (e.g., ICA, anterior and middle cerebral artery). Histopathological examination of the end of the carotid artery showed that the luminal stenosis was caused by fibrocellular intimal thickening, tortuosity, and disruption of internal elastic lam^50,51^. In recent years, the application of neuroimaging techniques such as high-resolution magnetic resonance imaging (MRI) in MMD patients has shown that the artery diameter of the involved segment is narrowed and the symptomatic segment is concentric enhanced^52–54^, consistent with previous findings of endometrial hyperplasia and medial thinning^55,56^. More evidences show that MMD is mainly an endometrial hyperplasia disease. The immunohistochemical features of the distal parts of ICAs indicated the proliferation of SMCs or ECs^8,51,57^. The migration and proliferation of SMCs associated with actin alpha 2 (*ACTA2)* mutations is considered to be a key mechanism of familial MMD^58^.

The second refers to the formation of unhealthy perforating arteries, the so-called moyamoya vessels, which are considered a compensation for cerebral ischemia and hypoxia^59^. Histopathological changes in moyamoya vessels include fibrin deposition in the vessel walls, elastic layer fragments, media weakening, and formation of micro artemia. Immunohistochemical studies have confirmed that many factors related to angiogenesis [VEGF receptors, fibroblast growth factor (FGF) receptor, nestin, and so on] were abnormally expressed in vascular ECs^51,60^, suggesting an active angiogenetic process. Although moyamoya vessels may supply the lack of perfusions, they are ineffective, fragile neovascularization that gradually disappear over time, leading to adult intracranial hemorrhage^61^. In conclusion, the excessive formation of collateral vessels that originated from the initial stenosis of the ICA emphasizes that the increase and/or abnormality of neovascularization are involved in the pathophysiological process of the disease^62^.

Moreover, various proangiogenesis cytokines have also been reported to be associated with MMD, including growth factors (such as VEGF, FGF, platelet-derived growth factor, and hepatocyte growth factor), cytokines related to vascular remodeling and angiogenesis (such as MMP and its inhibitors, hypoxia-inducible factor-1α and cell retinol node Syn-1), and inflammation-related cytokines^59^.

As described above, MMD is a special disease closely related to the dynamic between arterial proliferation and neovascularization, which may involve the proliferation, migration, differentiation, and maturation of vascular constituent cells and the maintenance of vascular structure. Therefore, the involvement of EPCs in MMD may be more complex than in other cerebrovascular diseases.

## Current Studies of EPCs in MMD

Since the first description of EPCs by Asahara et al. in 1997^21^, there have been a lot of studies on EPCs in various diseases, such as hypertension^63^, cardiovascular disease^64^, and cerebrovascular diseases^26^. Especially in cerebral ischemia stroke, EPCs-based cell therapy is now considered an important new therapeutic approach^65^. EPCs have also attracted attention in the pathogenetic study of MMD. The current studies mainly focused on the aspects given here (Table 1).

**Table 1.** Summary of Current Studies About Endothelial Progenitor Cells in Moyamoya Disease.

Authors	Nation	Year	Subjects	Sample source	Isolation and cultivation methods	Subsets (terminology) of EPCs	Criteria of characterization	Abnormal cell amount (MMD vs. HC)	Abnormal cell function (MMD vs. HC)
Yoshihara et al.	Japan	2008	4 MMD, 26 HC	Peripheral blood	A	cEPCs (circulating CD34^+^ cells)	CD34^+^CD45^+^	↑	
Jung et al.	Korea	2008	24 MMD, 48 HC	Peripheral blood	B	Early EPCs (EPC-CFU) and late EPCs (outgrowth cells)	1. Positive Ac-LDL uptake; 2. *Ulex europaeus* agglutinin-1, CD31^+^, vascular endothelium cadherin, CD34^+^, kinase domain receptor	1. EPC-CFU: ↓ 2. Outgrowth cells: ↑	Early EPCs: proliferation: ↓ Late EPCs: proliferation: ↑, tube formation: ↓
Rafat et al.	Germany	2009	20 MMD, 8 ACVD, 15 HC	Peripheral blood	A	cEPCs	CD34^+^/CD133^+^/VEGFR-2^+^	↑	
Kim et al.	Korea	2010	28 MMD, 12 HC	Peripheral blood	B	cEPCs in MMD children	For early EPC: cluster (central core of rounded cells surrounded by spindle-shaped cell), CD34^+^CD133^+^KDR^+^ For late EPC: vWF^+^, cobblestone morphology, positive Ac-LDL uptake	1. Early EPC and EPC clusters: ↓ 2. Outgrowth cells: ↓	Early EPCs: proliferation: ↓ Late EPCs: proliferation: ↓, tube formation: ↓, senescence: ↑
Ni et al.	China	2011	18 MMD, 12 HC	Peripheral blood	C	cEPCs	CD34^+^, CXCR4 (CD184)^+^	↑	CD34^+^CXCR4^+^ cells: ↑
Lee et al.	Korea	2015	9 MMD, 4 HC	Peripheral blood	B	Late EPCs (ECFCs)	CD34^+^KDR^+^CD133^+^CD31^+^, CD45^+^vWF^+^, positive Ac-LDL uptake		Tube formation: ↓
Zhang et al.	China	2016	30 MMD with STA-MCA, 27 MMD only conservative treatment	Peripheral blood	A	cEPCs	CD34^+^CD133^+^KDR^+^	The number of EPCs was decreased significantly after surgery	
Phi et al.	Korea	2017	12 MMD, 7 HC	Peripheral blood	B	Late EPCs (ECFCs)	CD34weakKDR^+^VE-cadherin^+^CD31^+^α-SMAweakPDGFR-α and βweak CD45^–^vWF^+^		1. Tube formation: ↓ 2. MMD ECFCs promote migration of SPCs
Choi et al.	Korea	2018	5 MMD, 5 HC	Peripheral blood	B	Late EPCs (ECFCs)	CD31^+^CD34^+^CD45^+^CD133^+^KDR^+^vWF^+^		1. Tube formation: ↓ 2. Disrupted mitochondrial morphology 3. Mitochondria functional abnormalities
Bao et al.	China	2018	66 MMD, 81 HC	Peripheral blood	C	cEPCs	CD31^+^CD45dimCD34brCD133^+^	↑	
Choi et al.	Korea	2018	Rat models	ECFCs from control/MMD patients were injected into the CCH rat model	B	Late EPCs (ECFCs)			1. Less improvement in the restoration of cerebral perfusion and in behavior 2. Less amount of neovasculogenesis and neurogenesis and more apoptosis

MMD: moyamoya disease; HC: healthy control; ACVD: atherosclerotic cerebrovascular disease; STA-MCA: superficial temporal; cEPCs: circulating endothelial progenitor cells; EPCs: endothelial progenitor cells; EPC-CFU: endothelial progenitor cells colony-forming unit; ECFCs: endothelial colony-forming cells; PDGFR: platelet derived growth factor recepto; SPC: smooth muscle progenitor cells; PBMNCs: peripheral blood mononuclear cells; Ac-LDL: acetylated low-density lipoprotein; CCH: chronic cerebral hypoperfusion; CFU-EC: colony-forming unit endothelial cells; VEGFR-2: vascular endothelial growth factor receptor-2; KDR: kinase insert domain receptor.

Isolation and culture methods:

A: Density gradient centrifugation to obtain PBMNCs and characterized by flow cytometry; B: density gradient centrifugation to obtain PBMNCs, culture 7days for EPC-CFU, characterized by flow cytometry; 2 months for outgrowth cells, characterized by flow cytometry; C: peripheral whole blood samples characterized by flow cytometry.

### EPCs Quantitative Anomaly

After acute cerebral ischemia, cluster differentiation 34 positive (CD34^+^) cells in the BM of stroke patients were activated^6^. In addition, transplantation of CD34^+^ cells^66^ and BM cells^67^ has been shown to restore cerebral blood flow in experimental stroke models. In chronic ischemia, CD34^+^ cell transplantation has also been shown to accelerate the formation of new blood vessels, including collateral vessels, in patients with chronic ischemic heart disease^68^ and limb ischemia^69^. In addition, there is a report on the relationship between the hypoplasia of coronary collateral and the decrease of circulating EPCs in patients with myocardial ischemia^70^. Thus, exploring the difference in the amount of EPCs between MMD patients and normal people may open the window to have a peep at the mechanisms of the complicated angiogenesis in MMD patients.

In 2008, Yoshihara et al.^71^ found for the first time that the number of CD34^+^ cells in the PB of MMD patients was significantly higher than that of normal people. Subsequently, researchers used more abundant molecular markers, such as CD133, vascular endothelial growth factor receptor-2 (kinase insert domain receptor) VEGFR-2 (KDR), and CD31, to characterize and count EPCs in PB. Similar results were observed^72–74^, except in Jung et al.^75^ and Kim et al.^76^ In these two studies, the researchers cultured the obtained peripheral blood mononuclear cells (PBMNCs) and subdivided the EPCs into early EPCs/endothelial progenitor cells colony-forming units (EPC-CFU) and late EPCs/outgrowth cells according to the morphological and molecular markers. Jung et al.^75^ found EPC-CFU numbers were significantly lower in MMD patients than in controls, while outgrowth cells were more in MMD patients. However, Kim et al.^76^ observed a decrease in both early EPCs and late EPCs. Because of this, the results of the amount of EPCs in the PB of MMD patients are often regarded as “controversial.”

### EPCs Functional Abnormality

As mentioned previously, EPCs can be divided into two subpopulations with great differences in morphology and capability. For early EPCs, one of the significant features is the ability to form clusters or colonies in in vitro cultivation. In particular, EPCs show clusters with spindle-shaped cells at the boundary^47^. Therefore, the formation of clusters and the number of these clusters are considered a definitive measure for evaluating EPCs numbers and differentiation^64^. As mentioned earlier, Jung et al.^75^ and Kim et al.^76^ both found early EPCs and clusters were significantly reduced in MMD patients compared with healthy control.

Late EPCs are closer to mature ECs in phenotype but show surprising tube-forming and proliferative capabilities, which are essential to promote neovascularization and maintain the integrity of vascular structure^47^. In all relevant studies^75–78^, the tube-forming ability of EPCs in MMD were decreased, but the results about proliferation function were debatable: Jung et al.^75^ observed outgrowth cells were more in MMD patients but Kim et al.^76^ observed outgrowth EPCs in MMD were less. Besides, in 2011, Ni et al.^73^ found a larger proportion of both CD34^+^ and C-X-C motif chemokine receptor 4 (CXCR4)-positive cells in the PB pool of EPCs in MMD patients than in healthy controls. CXCR4 is the receptor of SDF-1α, which interacts with SDF-1α for trafficking CD34^+^ cells or recruiting other vascular wall (progenitor) cells from BM to PB and modulating angiogenesis. Platelet-derived SDF-1α mediates the migration of CD34^+^ cells to the injured vessel and differentiate into EPCs via binding CXCR4^79^. Therefore, this has been considered as an indirect evidence of the enhanced migration ability of MMD-derived EPCs. However, Kim et al.^76^ found increased senescent-like phenotype of EPCs from pediatric MMD. Senescent EPCs have been found in impairments in multiple physiological activities, such as migration, differentiation, angiogenic activity, and alterations in growth factor expression^80–82^.

EPCs are described to contribute to neovascularization not only by differentiating into mature ECs but by paracrine effects, which stimulate angiogenic activity of resting mature ECs, leading to their migration, proliferation, and sprouting. Indeed, in 2017, Phi et al.^77^ confirmed C-C motif chemokine ligand 5 (CCL5) secreted by MMD endothelial colony-forming cells (ECFCs) significantly augmented the migration activities of SMCs (a main contributor to the hyperplasia of intima in MMD^8,57^) toward ECFCs.

In addition, retinaldehyde dehydrogenase 2 has been found downregulated in MMD EPCs and was attributed to defective acetyl-histone H3 binding to the promoter region^78^. The ECFCs from the MMD patients also displayed disrupted mitochondrial morphology like a shorter and more circular shape and functional abnormalities such as decreased oxygen consumption rate, increased intracellular Ca_2_^+^ concentration, and increased reactive oxygen species levels^83^. Except for the above in vitro experiments, the only relevant in vivo experiment found EPCs obtained from MMD brought less improvement in the cerebral perfusion, behavior, and amount of neovasculogenesis and neurogenesis after injection into the chronic cerebral hypoperfusion rat models^84^.

### Related Factors

Few factors related to the quantitative and functional abnormality of EPCs were found. Disease stage^75^, patient’s age^72,74^, and serum levels of VEGF^72^ were found to be inversely correlated to EPCs numbers. In addition, gene enrichment analysis showed the biological processes involving immune response and chemotaxis were significantly enhanced in MMD ECFCs, while biological processes related to cell cycle and deoxyribonucleic acid (DNA) repair were suppressed in MMD ECFCs. In metabolic and signaling pathways, the genes related to the chemokine signaling pathway, ECM–receptor interaction, and cell adhesion molecules were activated in MMD ECFCs, whereas the genes for DNA replication, cell cycle, and mismatch repair were downregulated^81^. Recently, Nagata et al.^85^ developed a method to investigated the characteristics of EPCs cultured from patients with MMD under conditions of activated anti-inflammatory and angiogenic monocytes/macrophages and concluded that insufficient production of interleukin 10 from M2 macrophages impairs EPCs differentiation in MMD patients.

## Issues About EPCs Study in MMD

EPCs are currently the most studied subtypes of different vascular progenitor cells. Most of these works are related to progenitor cells derived from PB and BM, and many publications show the contribution of EPCs to angiogenesis in tumorigenesis^86^, wound healing^20^, and ischemia^87^, as well as intimal re-endothelialization after vascular wall injury^88^. However, throughout the 21st century, the study of EPCs has become complicated and hindered by the separation, cultivation, and definition of different angiogenic cell subsets, which are all marked under the banner of EPCs but fail to comply with the necessary standards^13,89^. The research of EPCs in MMD also faces these problems:

### What Are EPCs—The Definition of EPCs

**Figure 1.**
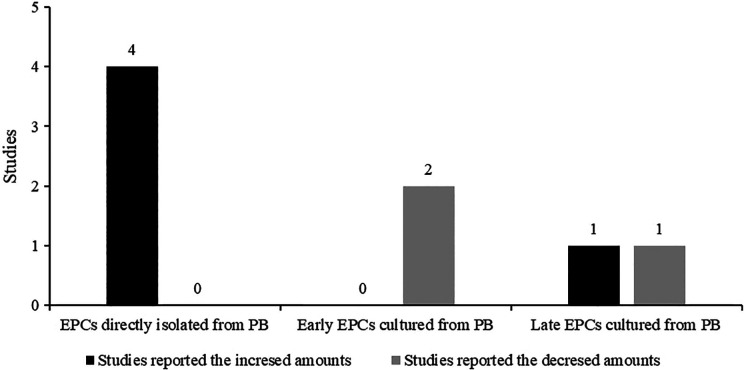
Different amounts of EPCs in moyamoya disease reported by different studies. EPCs: endothelial progenitor cells; PB: peripheral blood.

As shown in Table 1, there exist controversies in the quantity, survival, and functionality of the EPCs in different studies. For example, previous literatures all considered the results about the amount of EPCs in the PB of MMD patients were “controversial.” However, the controversial nature of those observations was actually the result of different classification methods and different isolation/cultivation strategies. As shown in Fig. 1, Yoshihara’s study and other three studies^71–74^ actually just observed the increased amounts of CD34^+^ or CD34^+^CD133^+^KDR^+^ or CD31^+^CD45^+^CD34^+^CD133^+^ cells directly obtained from PB of MMD patients, and their results were consistent. However, the investigated targets of the studies of Jung^75^ and Kim^76^ were “cultured” early EPCs and late EPCs from PBMNCs. Their results were consistent with early EPCs and opposite on late EPCs. Although all these studies claimed to be conducted in the name of EPCs, the results could not be simply summed up as “controversial” because they lack comparability. In fact, after we distinguished their results in terms of “early EPCs, “late EPCs,” and “EPCs directly from PB (without any further culture)” in Fig. 1, the quantity of EPCs in different studies became almost consistent. Accordingly, these studies only studied one subgroup of EPCs, and could not comprehensively reflect the exact situation of EPCs in the PB of MMD patients. Furthermore, different criteria (Table 1) of EPCs characterization also lead to inconsistent cell composition of “EPCs” in different studies, which may result in the inconsistent conclusions about EPCs cell function. Besides, the related studies are not so much in total, and there are also gaps in the sample size among studies, as well as the characteristics between the samples (such as the onset type, disease stage, age). These may also contribute to the bias in conclusions.

Unclear definitions also lead to inconsistent EPCs naming. In the past decade, various names/terms such as “circulating CD34^+^ cells,” “EPC-CFU,” “outgrowth cells,” “cEPCs,” “early EPCs,” “late EPCs,” colony-forming unit endothelial cells, and “ECFCs” have been adopted by different studies to describe EPCs. There exist inevitable reasons: the research pattern and methods of EPCs in MMD were almost based on researches of EPCs in other diseases such as cardiovascular disease and cerebral ischemic stroke. Even in those pioneering studies, unclear classification standards and lack of unified terms exist. Therefore, these phenomena are inevitable in the pathological study of EPCs in MMD. However, this situation has gradually improved. As shown in Table 1, researchers are gradually using relatively uniform terms and molecular markers to characterize EPCs. In order to solve the inconsistency/confusion of classification and names, at least two aspects must be achieved:

First, unified cell surface markers should be used to characterize EPCs and subgroups of EPCs. Cell surface markers are proteins and carbohydrates attached to cell membranes, providing a clear target for cell recognition. Various types of cell markers have been identified in the EPCs, such as CD34, a hematopoietic stem cell marker present in all types of ECs^90,91^. The pan-leukocyte marker CD45 is present only on EPCs but not on late EPCs or circulating ECs^47,90–92^. AC133/CD133 is expressed in HSCs and progenitor cells, early EPCs but not circulating ECs, indicating that prominin (mouse)-like 1 (AC133)/CD133 is an early marker^92^. On the other hand, there are conflicting reports about the expression of CD133 by late EPCs^91,93,94^. CD14 is a monocyte lineage marker; various studies have confirmed the presence of CD14 on early EPCs, but not on late EPCs and circulating ECs^49,95^. VEGFR-2 (mouse flk-1 or human KDR) is an important endothelial marker. VEGFR-2 expression in EPCs was weak, while VEGFR-2 expression was strong in late EPCs and ECs^47,49,96^. In addition, other markers such as CD36, CD106, and von Willebrand Factor (vWF) are rarely used in literature. Therefore, the true definition of different EPCs needs further study.

**Figure 2.**
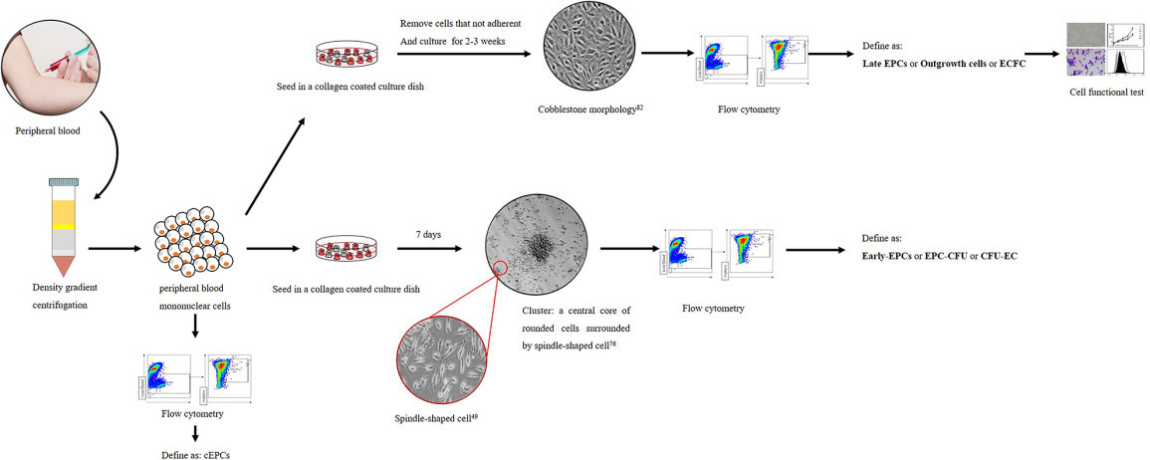
Methods of isolation, culture, and definition of circulation EPCs in moyamoya disease. EPCs: endothelial progenitor cells; cEPCs: circulation endothelial progenitor cells; EPC-CFU: endothelial progenitor cells colony-forming units; ECFCs: endothelial colony-forming cells; CFU-EC: colony-forming unit endothelial cells.

Second, unified isolation and culture methods are needed. We have summarized the isolation and cultivation methods of EPCs from the PB of MMD patients adopted by previous studies in Fig. 2. As show, different isolation and cultivation methods bring out different subsets of EPCs. Founded by Asahara et al. and developed by later researchers, a set of methods were used to isolate and cultivate the cEPCs. In general, after the PBMNCs are obtained from PB via density gradient centrifugation, they are inoculated into a collagen-coated culture dish. After a short period of culture, such as 7 days, clusters surrounded with spindle-like cells at the boundary form. These spindle-like cells are defined as early EPCs. If the PBMNCs are cultured for a longer period, such as 2–3 weeks, a “cobblestone” morphology will appear and these cells are referred to as outgrowth endothelial cells^97^, or late EPCs^47^, or ECFCs^98^. Collectively, these cells are termed as cEPCs. We also recommend techniques for isolating and culturing EPCs summarized by Chopra et al.^45^

### Why EPCs—Rationality About EPCs in the Pathogenesis of MMD

PB EPCs may contribute to MMD progression; however, other body parts in MMD patients show no obvious vascular atrophy. The microenvironment of brain may play a certain role in MMD. Neovascularization, which encompasses remodeling of existing vessels, angiogenesis, and barrier genesis, is a very complex process that requires coordination of cell-to-cell interaction^99^. This cellular communication is not limited to signals among vascular cells such as ECs–vascular SMCs or tip cells–stalk cells but indeed includes a network of vascular cells surrounding resident cells of the brain including pericytes, neurons, glia, and oligodendrocytes as well as circulating blood and BM cells^100^.

Recently, tissue-resident EPCs from large vessels have been considered as prime source for peripheral vascular repair because of their potential for significant cell proliferation, colony formation, drug excretion, and vascular formation^101,102^. Kawasaki et al. has demonstrated that the lung tissue–resident EPCs, rather than circulating EPCs, play a major role in pulmonary vascular repair of endotoxin-induced injury in the process of pulmonary vascular regeneration in experimental acute respiratory distress syndrome^103^. In fact, neuronal stem cells (NSCs) from human embryos have also been shown to express several endothelial and hematopoietic markers^104^. NSCs and peripheral nerve-derived adult pluripotent stem cells can be differentiated into ECs in vitro^105–107^. Studies using in vivo mouse models have shown that NSCs contribute to neurogenesis and angiogenesis not only in adult neurons but also in nonnerve tissues^105^. All of the above studies reflect two important findings: first, NSCs and ECs share a common progenitor cell; and second, the local environment is essential to control NSCs to ECs trans-differentiation.

In addition, EPCs may not be the only cells involved in neovascularization of patients with MMD. Aberrant angiogenesis in MMD is an active angiogenetic process that may recruit various cell types such as SMPCs^71^, SMCs^108^, circulating ECs^109^, and/or immune cells^110^ to cause both stenosis and abnormal collateral formation.

Another important question is the nature of moyamoya vessels. Are they “newly formed” perforator arteries? Or the remodeling and dilatation of preexisting vascular channels? The responsible cells and mechanisms of these two different “neovascularization” processes are different. So far, the “exact identity” of moyamoya vessels has not been determined. The biggest obstacles in the basic research of MMD are difficulty in obtaining specimens and the lack of animal model. Unlike other diseases, MMD has a relatively short history of discovery and research. Further study of its pathological mechanism needs to be based on solid, scientific, and abundant objective observation.

## Summary

Accurate and effective progenitor cell research provides a possible prospect for the exploration of the pathogenesis of MMD, but also faces many difficult challenges. More studies are needed to discover accurate mechanisms of EPCs mobilization, migration, (trans)differentiation, and homing to the target areas during the progress of MMD. Despite a large number of unsolved problems, more and more standardized scientific researches are providing some promising results. The aberrant EPCs amounts and impaired EPCs function may be related to the pathogenesis of MMD. However, improved and unified isolation, cultivation, and identification methods are needed to verify the rationality and feasibility of these results. Clinically, the formation of fragile compensatory vascular networks driven by abnormal neovascularization represents the source of cerebral hemorrhage in MMD patients. But at the same time, when using vascularized grafts such as temporal muscle to treat MMD, the expansion of angiogenesis is needed. Thus, a better understanding of the biology of EPCs will provide us with a clearer understanding of MMD and the possibility of early intervention, as well as to apply it to clinical therapy.
